# Anti-Plasmodial Activity of Some Zulu Medicinal Plants and of Some Triterpenes Isolated from Them

**DOI:** 10.3390/molecules181012313

**Published:** 2013-10-08

**Authors:** Mthokozisi B. C. Simelane, Addmore Shonhai, Francis O. Shode, Peter Smith, Moganavelli Singh, Andy R. Opoku

**Affiliations:** 1 Department of Biochemistry & Microbiology, University of Zululand, Private Bag X1001, KwaDlangezwa 3886, South Africa; E-Mails: adshon@gmail.com (A.S.); OpokuA@unizulu.ac.za (A.R.O.); 2 Department of Chemistry, University of Zululand, Private Bag X1001, KwaDlangezwa 3886, South Africa; E-Mail: francisshode@yahoo.com; 3 Division of Pharmacology, University of Cape Town, Private Bag X3, Rondebosch 7701, South Africa; E-Mail: Peter.Smith@uct.ac.za; 4 Discipline of Biochemistry, University of KwaZulu-Natal, Private Bag X54001, Durban 4000, South Africa; E-Mail: Singhm1@ukzn.ac.za

**Keywords:** *Plasmodium falciparum*, *Mimusops caffra*, *Mimusops obtusifolia*, *Hypoxis colchicifolia*, ursolic acid

## Abstract

*Mimusops caffra* E. Mey. ex A.DC and *Mimusops obtusifolia* Lam (both members of the *Sapotaceae* family), and *Hypoxis colchicifolia* Bak (family *Hypoxidaceae*) are used by traditional healers in Zululand to manage malaria. Anti-plasmodial investigation of the crude extracts and some triterpenes isolated from the plants showed activity against a chloroquine sensitive (CQS) strain of *Plasmodium falciparum* (D10)*.* Among the crude extracts the leaves of *M. caffra* exhibited the highest activity, with an IC_50_ of 2.14 μg/mL. The pentacyclic tritepenoid ursolic acid (**1**), isolated from the leaves of *M. caffra* was the most active compound (IC_50_ 6.8 μg/mL) as compared to taraxerol (**2**) and sawamilletin (**3**) isolated from the stem bark of *M. obtusifolia* (IC_50_ > 100). Chemical modification of the ursolic acid (**1**) to 3β-acetylursolic acid (**4**) greatly enhanced its anti-plasmodial activity. Compound **4** reduced parasitaemia against *Plasmodium berghei* by 94.01% in *in vivo* studies in mice. The cytotoxicity of 3β-acetylursolic acid (IC_50_) to two human cell lines (HEK293 and HepG2) was 366.00 μg/mL and 566.09 μg/mL, respectively. The results validate the use of these plants in folk medicine.

## 1. Introduction

Malaria is one of the major health problems in tropical Africa, South-east Asia, Central South America and Oceania. Despite the various efforts by governmental and non-governmental organizations aimed at eradicating the disease, malaria is said to kill a child every 30 s [1]. Malaria cases have been reported in other areas of the World that were previously considered eradicated of malaria [2].

In Africa, herbal medicines are an important part of the culture and traditions of its people [3]. Traditional healers use different concoctions prepared from medicinal plants to treat malaria. Given the remarkable anti-malarial properties of *Cinchona* bark that have been known for more than 300 years, resulting in the discovery of quinine [4] and the more recent development of artemisinin derivatives [5], the potential of plant species to provide effective drugs for the treatment of malaria cannot be overemphasized. Furthermore, the drug resistance of the malaria parasite to chloroquine and sulfadoxine–pyrimethamine, and also the toxicity of the currently available drugs have stimulated the search for alternative medicines which are naturally derived. In addition, modern health care to the rural people is still a far-reaching goal, due to economic constraints [6] and many vulnerable groups depend on plant-based traditional healing. The anti-malarial activity of many plants has been reported [7,8,9,10]. An ethonobotanical survey revealed the extensive utilization of *M. caffra*, *M. obtusifolia* and *H. colchicifolia* for the management of malaria in Zulu traditional medicine.

*M. caffra* is a small to medium-sized tree that grows up to 15 m high [11]. Its natural habitat is the dune forest from the high tide mark in KwaZulu-Natal and the Eastern Cape Provinces of South Africa and the Mozambique [11]. Medicinal uses of *M. caffra* include healing properties against sores and wounds [12].

*M. obtusifolia* has a small to medium-sized tree. Bark is grey to blackish, very rough and fissured in older specimens. Leaves are relatively broader and more rounded, 3.5–10 cm long. It is found in Southern Africa [13]. There is little information in the literature on its pharmacological activities.

*H. colchicifolia* is a slow-growing plant that often reaches up to 600 mm in height, with erect leaves; it is widespread in southern Africa [14]. It has a large underground tuber that allows it to survive the regular grass fires common to the grassland where it is found. These tubers are used by traditional healers to treat impotency and barrenness. Infusions are also taken as love charm emetics and are administered for hysterical fits [15]. Pharmacological activities of *H. colchicifolia* include anti-HIV and anti-diabetic properties [16,17]. This study was undertaken to investigate the anti-malarial activity of these plants.

## 2. Results and Discussion

Traditional medicines are a potential rich source of new drugs against malaria and other infectious diseases. The literature abounds with descriptions of the bioactivity of many antimalarial plants [7,8,9,10]. The observed anti-plasmodial activity of the crude extracts of *M. caffra*, *M. obtusifolia*, and *H. colchicifolia* is presented in Table 1. While the *Mimusops* species exhibited anti-plasmodial activity, it is apparent that our results do not support the traditional use of the bulb of *H. colchicifolia* in treating malaria. It is however worth mentioning that we observed antipyretic properties (data not included) of the extract of *H. colchicifolia*. Fever is the early symptom of malaria, and it is thus likely that the plant is used traditionally to treat the symptoms rather than the disease.

**Table 1:** Anti-plasmodial activity against *Plasmodium falcipurum* (CQS) D10 strain (*in vitro*) and *Plasmodium berghei* (*in vivo*).

Sample	^a^ IC_50_ (µg/mL)	^b^ Average % parasitemia	^b^ Average % suppression	^a^ Cytotoxicity (μg/mL)	
				HEK293	HepG2
*M. caffra* *(leaves)*	2.14	NT	NT		
*M. obtusifolia (bark)*	32.5	NT	NT		
*H. colchicifolia (bulb)*	NA	NT	NT		
Ursolic acid	6.8	NT	NT		
Ursolic acid acetate	1.9	0.07	94.01	366.00	566.09
3-oxo-ursolic acid	7.3	NT	NT		
Taraxerol	>100	NT	NT		
Sawamilletin	>100	NT	NT		
Artesunate	5.1 *	NT	NT		
Chloroquine	14.1 *	0.07	83.43		

* = ng/mL, ^a^ IC_50_ = Inhibitory concentration, ^b^ Average percentage parasitemia/suppression (*in vivo*), NT = not tested, NA = not active.

Several triterpenes have been reported to possess both *in vitro* and *in vivo* anti-plasmodial activity [18,19,20]. Of the three triterpenes that were isolated from *M. caffra* and *M. obtusifolia* (ursolic acid, taraxerol and sawamilletin), only ursolic acid showed any appreciable anti-plasmodial activity (IC_50_ 6.8 μg/mL) at the concentration tested. Ursolic acid has been previously reported to possess anti-plasmodial activity [21], and its presence in *M. caffra* could have contributed to the observed bioactivity of the plant. The lower activity of the extracted ursolic acid (compared to the crude *M. caffra* extract) could indicate a synergistic effect with other compounds, decomposition during fractionation, or removal of a protective matrix. Chemical modification of ursolic acid to its acetate derivative however resulted in a 72% increase in the *in vitro* anti-malarial activity (IC_50_ 1.9 μg/mL). Chemical modification of drugs has been known to improve the potency of the drugs [22,23]; it is noteworthy that the usoric acid acetate exhibits a 94.01% suppression of parasitemia in infected mice (*in vivo*).

In cytotoxic evaluations a compound is only considered significantly active with an IC_50_ of less than 30 μg/mL [24]. The cytotoxicity of ursolic acid acetate to HEK293 and HepG2 cell lines is presented in Table 1. It is apparent that, when dilution in the bloodstream is taken into account, ursolic acid acetate should be regarded as non-toxic. 

## 3. Experimental

### 3.1. Plant Collection

Fresh plant materials of *M. obtusifolia* and *H. colchicifolia* were collected from the Manguzi area, KwaZulu-Natal Province, South Africa, at the flowering stage in April, 2011 and *M. caffra* was collected in May 2012 from Durban, KwaZulu-Natal Province, South Africa. The plants were identified by Mrs. N. R. Ntuli, Department of Botany, University of Zululand, KwaDlangezwa. Voucher specimens were deposited at the University Herbarium [Simelane, MBC/02 (ZULU); Simelane, MBC/03 (ZULU); Simelane, MBC/04 (ZULU)].

### 3.2. Extraction and Isolation

The air-dried leaves of *M. caffra* (500 g) and the bark of *M. obtusifolia* (1.1 kg) were extracted with dichloromethane (DCM) and ethyl acetate (EtOAc) respectively (1:5 w/v). The resultant filtered extracts were concentrated to dryness under reduced pressure in a rotary evaporator (40 ± 2 °C). Dried extracts (5 g) were separately subjected to column chromatograph (20 × 500 mm) using silica gel 60 (300 g; 0.063 to 0.2 mm; 70 to 230 mesh ASTM supplied by Merck (Darmstadt, Germany). The crude extracts were chromatographed using gradient elution of hexane-ethyl acetate in a 5% stepwise increase at a speed of 100 mL per min and collecting 20 mL fractions. The collected fractions were combined based on their TLC (20 × 20 F254—Merck, Whitehouse Station, NJ, USA) profile to yield combined fractions. Visualization was achieved by UV light (254 nm) and by heating with 20% H_2_SO_4_ acid in MeOH. The single spot fractions were recrystallized in methanol and hexane to obtain 270 mg of MBCF93 from the *n*-hexane/EtOAc 7:3 eluate of the *M. caffra* extract; 310 mg of MBCF45 and 240 mg of MBCF15 were obtained from the *n*-hexane/EtOAc 9:1 fractions from the bark of *M. obtusifolia*. No attempt was made to isolate any compounds from *H. colchicifolia* because the initial crude extracts indicated no observable anti-plasmodial activity.

### 3.3. Structural Elucidation

**Figure 1:**
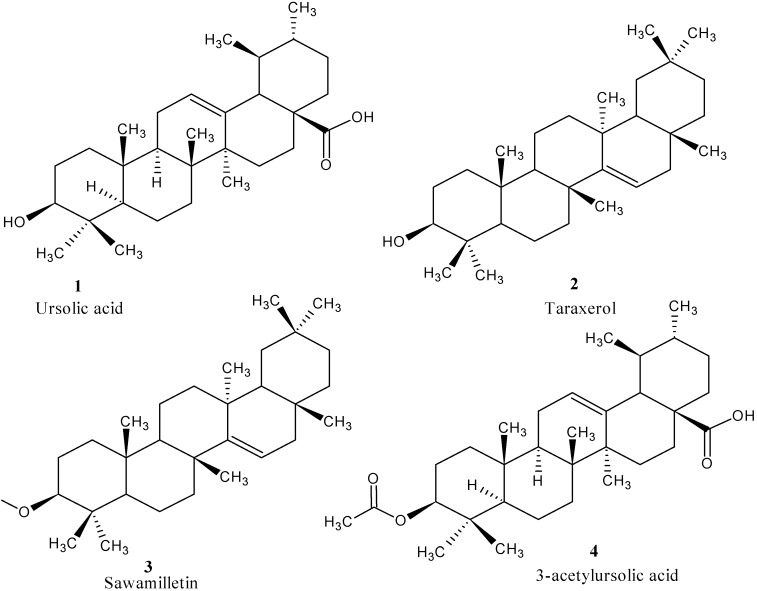
Chemical structures of the isolated triterpenes.

Compound MBCF93 was a white powder with a melting point (mp) of 279–283 °C, MBCF45 was also a white powder with mp 280–287 °C, MBCF15 also came out as a white powder with mp 272–285 °C. The structure of compounds were established using Nuclear Magnetic Resonance (NMR) techniques with the application of 2D-NMR (^1^H-^1^H, ^13^C-^13^C, DEPT, COSY, HMQC, HMBC and NOESY), infrared (IR) spectra and liquid chromatography mass spectrometry (LC-MS); molecular weights were identified by ESI-MS (positive mode). The compounds were confirmed by spectra comparison of their spectra (^1^H and ^13^C-NMR) with reported literature (Table 2). MBCF93 was confirmed as ursolic acid (**1**), MBCF45 as taraxerol (**2**), and MBCF15 as sawamilletin (**3**) (Figure 1).

**Table 2:** ^1^H- and ^13^C-NMR chemical shifts (δ, ppm) of compounds **1**, **4** and **2**.

Carbon Position	UA(1)	δ ^1^H (ppm)			DEPT	UAA(4)	DEPT
	δ ^13^C (ppm)					δ ^13^C (ppm)	
1	38.7				CH_2_	38.3	CH_2_
2	23.5				CH_2_	24.1	CH_2_
3	79	3.43 (1H, brs)			CH	80.9	CH
4	39.6				C	37.7	C
5	52.7				CH	55.3	CH
6	18.3				CH_2_	18.2	CH_2_
7	33				CH_2_	32.9	CH_2_
8	39.1				C	39.5	C
9	47.6				CH	47.9	CH
10	36.7				C	36.7	C
11	23.7				CH	23.3	CH
12	125.8	5.50 (1H, brs)			CH	125.8	CH
13	138				C	138	C
14	42				C	41.9	C
15	29.4				CH_2_	30.6	CH_2_
16	23.3				CH_2_	23.6	CH_2_
17	47.9				C	47.5	C
18	55.3	2.52 (1H, d, *J* = 11.0 Hz)			CH	52.6	CH
19	30.6				CH	39	CH
20	30.4				CH	38.8	CH
21	27.3				CH_2_	30.6	CH_2_
22	37				CH_2_	36.9	CH_2_
23	23.4	1.24 (3H, s)			CH_3_	23.6	CH_3_
24	17	1.02 (3H, s)			CH_3_	17.1	CH_3_
25	17	0.93 (3H, s)			CH_3_	16.7	CH_3_
26	15.5	1.05 (3H, s)			CH_3_	17.1	CH_3_
27	24.2	1.22 (3H, s)			CH_3_	21.3	CH_3_
28	176				C	182.6	C
29	21.1	0.97 (3H, s)			CH_3_	15.5	CH_3_
30	23.4	0.99 (3H, d, *J* = 6.1 Hz)			CH_3_	21.2	CH_3_
-COCH_3_						28.1	
-COCH3						171	
**Carbon Position**	**Taraxerol (2) δ ^13^C (ppm)**		**DEPT**	**δ ^1^H (ppm)**			
1	38		CH_2_				
2	27.2		CH_2_				
3	79.1		CH				
4	39		C				
5	55.6		CH				
6	18.8		CH_2_				
7	35.1		CH_2_	2.0 (1H, dt, *J* = 3.1, 12.6 Hz, H-7a)			
8	38.8		C				
9	48.8		CH				
10	37.6		C				
11	17.5		CH_2_				
12	35.8		CH_2_				
13	37.6		C				
14	158.1		C				
15	116.9		CH	5.5 (1H, dd, *J* = 3.2, 8.2 Hz)			
16	36.7		CH_2_	1.9 (1H, dd, *J* = 3.0, 14.6 Hz, H-16a)			
17	37.7		C				
18	49.3		CH				
19	41.3		CH_2_				
20	28.8		C				
21	33.7		CH_2_				
22	33.1		CH_2_				
23	28		CH_3_	0.98 (3H, s, H-23)			
24	15.4		CH_3_	0.80 (3H, s, H-24)			
25	15.5		CH_3_	0.93 (3H, s, H-25)			
26	29.8		CH_3_	1.09 (3H, s, H-26)			
27	25.9		CH_3_	0.91 (3H, s, H-27)			
28	29.9		CH_3_	0.82 (3H, s, H-28)			
29	33.4		CH_3_	0.95 (3H, s, H-29)			
30	21.3		CH_3_	0.90 (3H, s, H-30)			

### 3.4. Acetylation of Ursolic Acid

The plant derived ursolic acid (283 mg) was treated with acetic anhydride (10 mL) and pyridine (20 mL). The mixture was stirred at room temperature overnight after which water (10 mL) was added and stirred for at least 30 min. The resultant solid was then filtered and washed thoroughly with dilute HCl solution under vacuum. The white powder obtained (300 mg, mp 280–282 °C) was identified, by its spectral properties, as 3β-acetylursolic acid (**4**).

### 3.5. Preparation of 3-Oxoursolic Acid (**5**)

**Figure 2:**
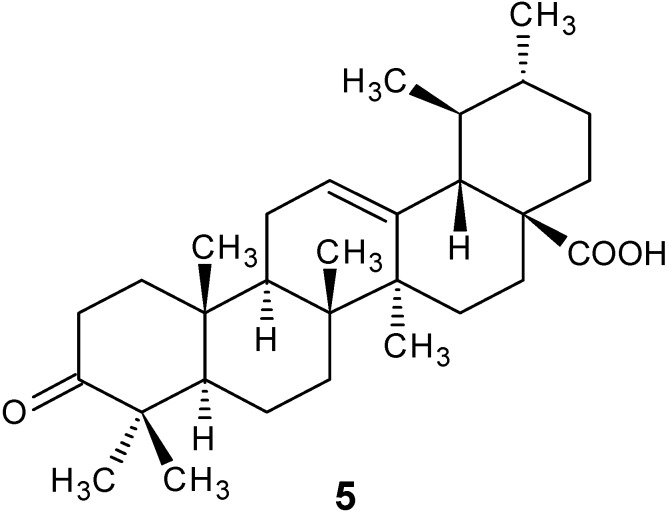
Chemical structure of 3-oxourosolic acid.

To a solution of compound 4 (100 mg) in acetone (1.5 mL) Jones’ reagent (0.4 mL) was added dropwise in an ice-salt bath. The reaction mixture was allowed to warm up to room temperature and stirred for 1 h. After cooling to 0 °C, 2-propanol (5 mL) was added and the solution stirred at room temperature for 30 min. The green precipitate was collected and washed well with acetone. The acetone solution from the combined filtrates were concentrated and dried. By purification on a silica gel column compound **5** was obtained as a white solid (86 mg) and identified as 3-oxo-ursolic acid (Figure 2).

### 3.6. Drug Sensitivity Assay

#### 3.6.1. *In Vitro* Antiplasmodial Activity

The test samples (crude extracts, isolates, and chemical derivatives) were tested in triplicate against a chloroquine sensitive (CQS) strain of *Plasmodium falciparum* (D10). Continuous *in vitro* cultures of asexual erythrocyte stages of *P. falciparum* were maintained using modified method of Trager and Jensen [25]. Quantitative assessment of anti-plasmodial activity *in vitro* was determined via the parasite lactate dehydrogenase assay using a modified method described by Makler [26]. The test samples were prepared to a 20 mg/mL stock solution in 100% DMSO and sonicated to enhance solubility. Stock solutions were stored at −20 °C. Further dilutions were prepared on the day of the experiment. Chloroquine and artesunate were used as the reference drugs for experiments. A full dose-response was performed for all active compounds to determine the concentration inhibiting 50% of parasite growth (IC_50_-value). Test samples were tested at a starting concentration of 100 μg/mL, which was then serially diluted 2-fold in complete medium to give 10 preparations of variable concentrations; with the lowest concentration being 0.2 μg/mL. The same dilution technique was used for all samples. CQ was tested at a starting concentration of 1,000 ng/mL. The highest concentration of solvent to which the parasite-infected erythrocytes were exposed to had no measurable effect on the parasite viability (data not shown). The IC_50_-values were obtained using a non-linear dose-response curve fitting analysis via Graph Pad Prism v.4.0 software (Graph Pad Prism, Inc: San Diego, CA, USA, 1994–2003).

#### 3.6.2. *In Vivo* Antiplasmodial Activity

This study was carried out after the approval from the Ethical Committee on Animal Use and Care of the University of Zululand (UZREC 171110-030 PGD 2013/26). Swiss mice (20–25 g each) of both sexes were obtained from biomedical research unit (BRU) in the University of KwaZulu Natal, Durban. They were kept in plastic cages, and given standard laboratory diet and water *ad libitum* and maintained under laboratory conditions of temperature and 12 h light and 12 h dark cycle. The animals were allowed to acclimatize to the laboratory at a controlled temperature of 22 °C for 15 days before being subjected to the experiments. The animals were divided into seven groups (eight in each group) consisting of control, chloroquine (5 mg/kg/day), and the extract treated group. The extract treated group was sub divided into five sub groups that received 50, 100, 200, 300 and 400 mg/kg/day body weight respectively.

##### 3.6.2.1. Parasite Inoculation

A single donor mouse infected with *Plasmodium berghei* parasites was bled into sterile heparinized culture medium and the blood was diluted with RPMI 1640 medium. The healthy experimental mice were infected intravenously via a tail vein with 0.2 mL of the diluted blood containing 1 × 10^7^ parasitized (*Plasmodium berghei*) red blood cells on day one.

##### 3.6.2.2. Evaluation of Antimalarial Activity

The antimalarial activity tests were performed using the four-day suppressive tests described by Peters *et al*. [27]. The extracts dissolved in 0.5% carboxymethyl cellulose (CMC) were administered orally using cannula (equivalent to 0.2 mL solution per mouse) for four consecutive days. Parallel tests with chloroquine were conducted for reference purposes at the same dose in one group and with an equivalent volume of 0.5% CMC (0.2 mL/mouse/day) in the control group. Thin smears were obtained from the tail vein of each mouse on day five after infection. The smears were fixed with methanol and stained with Giemsa stain. The percent parasitemia suppression was determined by counting the number of parasitized erythrocytes out of 500 red blood cells on random fields under the microscope. The average percentage suppression of parasitemia was calculated using the following formula:

% suppression = A – B/C
(1)

where A = % parasitemia in untreated controls, B = % parasitemia in treated groups, and C = % parasitemia in untreated controls. The data were analyzed using the F-test. A *P*-value < 0.05 was considered significant.

### 3.7. MTT Cell Proliferation Assay

Human embryonic kidney (HEK293) and human hepatocellular carcinoma (HepG2) cells were all grown to confluenecy in 25 cm^2^ flasks. This was then trypsinized and plated into 48 well plates at specific seeding densities. Cells were incubated overnight at 37 °C. Medium was then removed and fresh medium (MEM + Glutmax + antibiotics) was added. Extracts (50–350 μL/mL) were then added in triplicate and incubated for 4 h. Thereafter medium was removed and replaced by complete medium (MEM + Glutmax + antibiotics + 10% Fetal bovine serum). After 48 h cells were subjected to the MTT assay [28]. Data were evaluated through regression analysis using QED statistics program and from the linear equation the IC_50_ values representing the lethal concentration for 50% mortality was calculated.

### 3.8. Statistical Analyses

The mean and standard error mean of three experiments were determined. Statistical analysis of the differences between mean values obtained for experimental groups were calculated using Microsoft Excel Program, 2010 and Graph Pad Prism v.4.0 software (Graph Pad Prism, Inc: San Diego, CA, USA, 1994–2003) for IC_50_. Data were subjected to one way analysis of variance (ANOVA). *P* values < 0.05 were regarded as significant and *P* values < 0.01 as very significant.

## 4. Conclusion

This study was conducted to investigate the anti-malarial activity of some indigenous plants used by Zulu traditional healers to treat malaria. Even though the observed activity of the crude plant extracts, the isolated triterpenes, and the chemical derivative may not be as high as those reported for the standards (chloroquine and artesunate), the activity of the extracts were dose dependent, and with low toxicity levels, encourages the use of *M. caffra* in managing malaria in traditional medicine.
